# Analysis of *Lactobacillus* Products for Phages and Bacteriocins That Inhibit Vaginal Lactobacilli

**DOI:** 10.1155/S1064744997000410

**Published:** 1997

**Authors:** Lin Tao, Sylvia I. Pavlova, Susan M. Mou, Wen-ge Ma, Ali O. Kiliç

**Affiliations:** 1 Department of Oral Biology School of Dentistry University of Missouri-Kansas City 650 East 25th Street Kansas City MO 64108 USA; 2 Department of Obstetrics and Gynecology School of Medicine University of Missouri-Kansas City Kansas City MO USA; 3 Department of Microbiology and Clinical Microbiology School of Medicine Karadeniz Technical University Trabzon Turkey

## Abstract

*Objective:* Bacterial vaginosis is associated with an unexplained loss of vaginal lactobacilli. Previously, we have identified certain vaginal lactobacilli-released phages that can inhibit in vitro other vaginal lactobacilli. However, there is no apparent route for phages to be transmitted among women. The purpose of this study was to identify whether certain *Lactobacillus* products commonly used by women release phages or bacteriocins that can inhibit vaginal lactobacilli.

*Methods:* From 26 *Lactobacillus* products (2 acidophilus milks, 20 yogurts, 3 *Lactobacillus* pills, and 1 vaginal douche mix), lactobacilli were isolated with Rogosa SL agar (Difco, Detroit, MI). From these lactobacilli, phages and bacteriocins were induced with mitomycin C and tested against a collection of vaginal *Lactobacillus* strains.

*Results:* From the 26 products, 43 *Lactobacillus* strains were isolated. Strains from 11 yogurts released phages, among which 7 inhibited vaginal lactobacilli. Eleven strains released bacteriocins that inhibited vaginal lactobacilli. While about one-half of the vaginal strains were lysed by bacteriocins, less than 20% were lysed by phages.

*Conclusions:* Some vaginal lactobacilli were inhibited in vitro by phages or bacteriocins released from *Lactobacillus* products used by women, implying that vaginal lactobacilli may be reduced naturally due to phages or bacteriocins from the environment.


Infectious Diseases in Obstetrics and Gynecology 5:244–251 (1997)
© 1997 Wiley-Liss, Inc.

### Analysis of *Lactobacillus* Products for Phages and Bacteriocins That Inhibit Vaginal Lactobacilli

Lin Tao,[1]* Sylvia I. Pavlova,[1] Susan M. Mou,[2] Wen-ge Ma,[1] and Ali O. Kiliç[3]

[1]*Department of Oral Biology, School of Dentistry, University of Missouri-Kansas City, Kansas City, MO*

[2]*Department of Obstetrics and Gynecology, School of Medicine, University of Missouri-Kansas City, Kansas City, MO*

[3]*Department of Microbiology and Clinical Microbiology, School of Medicine, Karadeniz Technical University, Trabzon, Turkey*

ABSTRACT

*Objective:* Bacterial vaginosis is associated with an unexplained loss of vaginal lactobacilli. Previously, we have identified certain vaginal lactobacilli-released phages that can inhibit in vitro other vaginal lactobacilli. However, there is no apparent route for phages to be transmitted among women. The purpose of this study was to identify whether certain *Lactobacillus* products commonly used by women release phages or bacteriocins that can inhibit vaginal lactobacilli.

*Methods:* From 26 *Lactobacillus* products (2 acidophilus milks, 20 yogurts, 3 *Lactobacillus* pills, and 1 vaginal douche mix), lactobacilli were isolated with Rogosa SL agar (Difco, Detroit, MI). From these lactobacilli, phages and bacteriocins were induced with mitomycin C and tested against a collection of vaginal *Lactobacillus* strains.

*Results:* From the 26 products, 43 *Lactobacillus* strains were isolated. Strains from 11 yogurts released phages, among which 7 inhibited vaginal lactobacilli. Eleven strains released bacteriocins that inhibited vaginal lactobacilli. While about one-half of the vaginal strains were lysed by bacteriocins, less than 20% were lysed by phages.

*Conclusions:* Some vaginal lactobacilli were inhibited in vitro by phages or bacteriocins released from *Lactobacillus* products used by women, implying that vaginal lactobacilli may be reduced naturally due to phages or bacteriocins from the environment. Infect. Dis. Obstet. Gynecol. 5:244–251, 1997. © 1997 Wiley-Liss, Inc.

KEY WORDS

yogurt; *Lactobacillus* pills; vaginal douche mix; bacteriophages

While lactobacilli can be quite common and inconsequential environmental bacteria, lactobacilli indigenous to the human vagina are beneficial to women's health.[1] These bacteria normally produce lactic acid that maintains a pH of 4.0–4.5 and thus prevents vaginal infections caused by other microorganisms. However, an unexplained decrease of vaginal lactobacilli occurs during bacterial vaginosis (BV).[2] Women who suffer from BV may have an increased, milk-like discharge that has an unpleasant odor[3] and an increased risk to develop pelvic infections or prema-

Contract grant sponsor: Concerned Parents for AIDS Research/American Foundation for AIDS Research; Contract grant number: 02069-15-RG. Contract grant sponsor: University of Missouri Research Board; Contract grant number: K-3-40532.
*Correspondence to: Dr. Lin Tao, Department of Oral Biology, School of Dentistry, University of Missouri-Kansas City, 650 East 25th Street, Kansas City, MO 64108. E-mail:taol@smtpgate.umkc.edu

Received 16 October 1996
Accepted 23 June 1997

Clinical Study ture labor during pregnancy.[4] Although BV is a common vaginal infection, its cause is unknown. Women who are sexually active appear to be at a higher risk for BV, but BV has not been confirmed to be a sexually transmitted disease because it also occurs among women who are not sexually active.[3] Recently, BV has been defined as a condition in which the normal *Lactobacillus*-predominant flora is replaced with anaerobic bacteria, *Gardnerella vaginalis*, and *Mycoplasma hominis*.[4] Because the anaerobic bacteria are sensitive to hydrogen peroxide and lactic acid produced by vaginal lactobacilli, anaerobes theoretically should not outnumber lactobacilli.[5–7] Therefore, during the initiation of BV, vaginal lactobacilli might decrease first, thus allowing anaerobes to overgrow.

Several possible mechanisms by which vaginal lactobacilli decrease have been proposed. These include douching,[8] the use of spermicide, such as Nonoxynol 9,[9] and treatment with antibiotics for other infections. It is unknown, however, whether vaginal lactobacilli could decrease by natural causes. Previously, we have reported that some vaginal lactobacilli are lysogens that release phages, the viruses that infect bacteria.[10] These phages could infect many vaginal lactobacilli in vitro. This observation suggested that vaginal lactobacilli can be inhibited naturally by phages released from other vaginal lactobacilli. However, there is no apparent route for infective phages to be transmitted among women. Therefore, if phages are involved in the natural reduction of vaginal lactobacilli, which might occur frequently because BV is common, alternative sources of phages that are exposed to women may exist. One such source could be the environment, such as *Lactobacillus*-containing foods, vaginal douches, or suppository tablets.

Various products containing lactobacilli, such as yogurt, acidophilus milk, vaginal suppository tablets or capsules, and vaginal douche mix, have been used by women to treat vaginal infections.[5,11] Although the method of delivery varies—ingestion, vaginal instillation, or douching—the effects of the products have been inconsistent.[12] To test the hypothesis that phages or bacteriocins, which are antibacterial agents produced by bacteria, from the environment may inhibit vaginal lactobacilli, we analyzed whether some *Lactobacillus* products, through the release of phages or bacteriocins, could inhibit endogenous vaginal lactobacilli in vitro.

### MATERIALS AND METHODS

Twenty-six *Lactobacillus* products were purchased at local pharmacies, grocery stores, and health-food establishments. These products included 2 acidophilus milks, 20 yogurts, 3 *Lactobacillus* pills, and 1 *Lactobacillus* vaginal douche mix. The brand names and manufacturers of these products are listed in Table 1.

To isolate *Lactobacillus* strains from these products, a loop full of each product was streaked on the Rogosa SL agar (Difco, Detroit, MI) and incubated in a candle jar at 37°C for 48 h. Each *Lactobacillus* isolate was identified on the basis of growth on Rogosa SL agar, gram-positive staining, rod cell morphology, and catalase-negative reaction. *Lactobacillus* species were tentatively identified according to their sugar fermentation patterns compared with the scheme described in Bergey's Manual[13] and by an assay based on polymerase chain reaction (PCR) developed in our laboratory (unpublished data). From these *Lactobacillus* isolates, both bacteriophages and bacteriocins were induced by the mitomycin C method.[14] Briefly, 0.1 ml of overnight *Lactobacillus* culture in MRS broth (pH 5.5; Difco) was transferred into 10 ml prewarmed fresh MRS broth. After 3 h of incubation, the culture was divided equally into two test tubes. One tube was used as control, and the other had mitomycin C (Sigma, St. Louis, MO) added at a final concentration of 0.2 μg/ml. The induction of *Lactobacillus* phages or bacteriocins was indicated by a rapid reduction of optical density of the culture 5–7 h after the addition of mitomycin C. The lysates were centrifuged and filtered to remove unlysed cells and maintained at 4°C. Each lysate was tested for its inhibitory effect against a collection of 37 human vaginal *Lactobacillus* strains, which were isolated from 27 women (1 Native American, 5 Blacks, 5 Asians, and 16 Caucasians).[10]

Bacteriophages were differentiated from bacteriocins by the following procedures: 1) phage plaque assay; 2) isolation of phage DNA with the Qiagen kit (Qiagen, Chatsworth, CA); 3) hybridization of *Lactobacillus* chromosomal DNA with a nonradioactive biotin-labeled *Lactobacillus* phage DNA probe (Life Technologies, Gaithersburg, MD); and

TABLE 1. *Lactobacillus* products analyzed in this study

| Product[a] | Manufacturer | Location |
|---|---|---|
| Acidophilus milk | | |
| Anderson Erickson | Anderson Erickson Dairy Co. | Des Moines, IA 19053 |
| Fairmont-Zarda | Fairmont-Zarda Div. Roberts Dairy Co. | Omaha, NE 68101 |
| Yogurt | | |
| Alta Dena | Alta Dena Certified Dairy, Inc. | Noustry, CA 91744 |
| Always Save | Associated Wholesale Grocers, Inc. | Kansas City, KS 66106 |
| Anderson Erickson | Anderson Erickson Dairy Co. | Des Moines, IA 19053 |
| Belfonte | Belfonte Ice Cream Co. | Kansas City, MO 64127 |
| Best Choice Lite | Associated Wholesale Grocers, Inc. | Kansas City, KS 66106 |
| Breyers | Kraft General Foods, Inc. | Glenview, IL 60025 |
| Cascadefresh | Cascadefresh, Inc. | Seattle, WA 98125 |
| Colombo | Colombo, Inc. | Minneapolis, MN 55440 |
| Dannon | Dannon | Jacksonville, FL 32231 |
| Dillons | Dillon's Store Div. of Dillon Co., Inc. | Hutchinson, KS 67501 |
| Fairmont-Zarda | Fairmont-Zarda Dairy Co. | Kansas City, MO 64128 |
| Great Value | Wal*Mart Stores, Inc. | Bentonville, AR 72716 |
| Horizon Organic | Natural Horizon, Inc. | Boulder, CO 80301 |
| Mountain High | Mountain High, Inc. | Englewood, CO 80110 |
| Schnucks Lite | Schnuck Market, Inc. | St. Louis, MO 63106 |
| TCBY | Polytainers, Inc. | Little Rock, AR 72201 |
| Weight Watcher | Weight Watcher International, Inc. | Pittsburgh, PA 15230 |
| Wells' Blue Bunny | Wells' Dairy, Inc. | LeMars, IA 51031 |
| Yonson | Favorite Foods, Inc. | Fullerton, CA 92631 |
| Yoplait | Yoplait USA, Inc. | Minneapolis, MN 51031 |
| *Lactobacillus* pill | | |
| Lactinex | Becton-Dickinson Microbiology Sys. | Cockeysville, MD 21030 |
| Nature's Plus | Natural Organics, Inc. | Melville, NY 11747 |
| Solaray | Solaray, Inc. | Ogden, UT 84403 |
| *Lactobacillus* douche | | |
| Hygenia | Schiff Products, Inc. | Moonachie, NJ 07074 |

[a]The strains isolated from the 2 acidophilus milks were designated A1 and A2. The strains isolated from the 20 yogurts were designated Y1–Y20. The strains isolated from the *Lactobacillus* pills and vaginal douche mix were designated L1–L4. To protect the reputation of these commercial products, the sources of the phages and bacteriocins are not specifically indicated.

4) observation of the phages under an electron microscope.[15] Additionally, phage DNA fingerprinting analysis with restriction enzyme digestion and subsequent electrophoresis[16] was used to identify whether the isolated phages were genetically related. If no phages were identified from a mitomycin C-induced lysate, its inhibitory effect was analyzed for *Lactobacillus* bacteriocins. Catalase was added and pH was neutralized to rule out the effects of hydrogen peroxide and acid, respectively. The lysate was also heated at 100°C for 10 min and treated with proteolytic enzymes (trypsin, pepsin, and protease) to characterize the bacteriocins.

The assay for phage infection or bacteriocin inhibition was performed as described previously.[15] Briefly, an aliquot of *Lactobacillus* indicator culture at mid-exponential growth phase was mixed with soft agar (48°C) made of the *Lactobacillus* MRS medium supplemented with 10 mM $CaCl_2$ (MRS-C) and poured onto an MRS-C agar plate. The induced lysates were dropped onto the solidified soft agar, and the plates were incubated for 24 h at 37°C. The phage infection or bacteriocin lysis was indicated by a clear lysis zone in the soft agar layer. To extend the range of *Lactobacillus* species and strains beyond those isolated from the 27 patients and the 26 *Lactobacillus* products, an additional 11 *Lactobacillus* strains were also tested. These included 7 strains from American Type Culture Collection (ATCC; Rockville, MD), 3 strains from National Collection of Dairy Organisms (NCDO; Reading, England), and 1 strain from Dr. Klaenhammer (North Carolina State University, Raleigh). The 3 human strains (1 intestinal strain, *L. gasseri* ADH; and 2 vaginal strains, *L. gasseri* ATCC 9857 and *L. jensenii* ATCC 25258) were used as additional indicator strains, while the 8 dairy strains (*L. delbrueckii* subsp. *bulgaricus* NCDO 1489, ATCC

TABLE 2. Inhibition of exogenous *Lactobacillus* strains on vaginal lactobacilli by releasing phages or bacteriocins in vitro

| Exogenous effector strain[a] | No. of total sensitive strains (N/41) | Indicator strain[b] | | | | | | |
|---|---|---|---|---|---|---|---|---|
| | | *La* (3) | *Lg* (10) | *Lc* (18) | *Lj* (5) | *Lp* (1) | *Lf* (3) | *Ldl* (1) |
| Phage releaser | | | | | | | | |
| Y15 | 7 | 2 | 1 | 1 | 2 | 1 | 0 | 0 |
| Y8 | 5 | 2 | 1 | 1 | 0 | 0 | 0 | 1 |
| Y16 | 3 | 0 | 1 | 0 | 1 | 0 | 0 | 1 |
| Y1, Y10, Y11 | 2 | 0 | 1 | 0 | 0 | 0 | 0 | 1 |
| Y19 | 2 | 0 | 1 | 1 | 0 | 0 | 0 | 0 |
| Y2, Y5, Y12a, Y13 | 1 | 0 | 0 | 0 | 0 | 0 | 0 | 1 |
| Bacteriocin producer | | | | | | | | |
| NCDO 1489 | 19 | 0 | 1 | 15 | 2 | 1 | 0 | 0 |
| L1 | 11 | 3 | 1 | 1 | 4 | 1 | 0 | 1 |
| NCDO 2395 | 10 | 2 | 1 | 1 | 4 | 1 | 0 | 1 |
| Y12b | 10 | 2 | 2 | 0 | 5 | 0 | 0 | 1 |
| L2 | 7 | 0 | 0 | 5 | 1 | 0 | 0 | 1 |
| Y3 | 6 | 0 | 1 | 3 | 1 | 0 | 0 | 1 |
| Y17 | 3 | 0 | 0 | 3 | 0 | 0 | 0 | 0 |
| Y14 | 2 | 0 | 2 | 0 | 0 | 0 | 0 | 0 |
| Y18 | 2 | 0 | 1 | 0 | 1 | 0 | 0 | 0 |
| A1, A2, Y6 | 1 | 0 | 0 | 0 | 0 | 0 | 0 | 1 |
| Y7, Y9, Y20, L4 | 1 | 0 | 1 | 0 | 0 | 0 | 0 | 0 |

[a]Strains that gave the same results are listed in the same line.
[b]Vaginal *Lactobacillus* species: *La, L. acidophilus*; *Lg, L. gasseri*; *Lc, L. crispatus*: *Lj, L. jensenii*; *Lp, L. plantarum*; *Lf, L. fermentum*; *Ldl, L. delbrueckii* subsp. *lactis* ATCC 15808. The total number of strains of each species is indicated in parentheses.

11842, ATCC 27558, and ATCC 9649; *L. delbrueckii* subsp. *lactis* ATCC 15808; *L. helveticus* NCDO 87, NCDO 2395, and ATCC 15009) were used as additional effector strains.

Electron microscopy was performed as described previously.[10] Briefly, one drop of the purified phage ϕy8 in 0.1 M ammonium acetate (pH 7.0) was spotted on grids with a carbon-coated Formvar film (Ladd Research Industries, Inc., Burlington, VT). After drying for 30 sec, the sample was negatively stained with 2% uranyl acetate (pH 4.2) and observed under a CM12 Philips transmission electron microscope (Philips Electronic Instruments, Inc., Mahwah, NJ) at 80 kV.

### RESULTS

Among 26 products, 43 *Lactobacillus* strains were isolated with some products containing multiple *Lactobacillus* strains. According to their sugar fermentation patterns,[13] sensitivity patterns to a collection of dairy *Lactobacillus* phages and bacteriocins, plasmid profiles and phage contents, and the PCR assay based on their 16S RNA gene sequence (data not shown), the isolates were tentatively identified as the following species: *L. acidophilus* (23 strains divided into 2 groups: A, 10 strains with a 6 kb cryptic plasmid; and B, 13 strains without a plasmid), *L. helveticus* (2 strains), *L. delbrueckii* subsp. *bulgaricus* (13 strains), *L. delbrueckii* subsp. *lactis* (2 strains), and *L. plantarum* (3 strains). On the other hand, the human vaginal *Lactobacillus* species used as indicator strains included: *L. acidophilus* (3 strains), *L. crispatus* (18 strains), *L. fermentum* (3 strains), *L. gasseri* (9 strains), *L. jensenii* (5 strains), and *L. plantarum* (1 strain). Also included as indicators were a human intestinal strain *L. gasseri* ADH and a dairy strain *L. delbrueckii* subsp. *lactis* ATCC 15808. The latter has been commonly used as a phage indicator strain in *Lactobacillus* phage studies because it is susceptible to a broad range of *Lactobacillus* species and could show clear phage plaques.[10]

In the phage infection and bacteriocin lysis experiment, a collection of 50 induced lysates (43 from the 26 *Lactobacillus* products and 7 from the dairy type strains) was used to cross-interact with the 41 (39 vaginal, 1 intestinal, and 1 dairy) indicator strains. The results are shown in Table 2. While about one-half of the human vaginal strains displayed bacteriocin-induced lysis, less than 20% were sensitive to the dairy phages. By phage

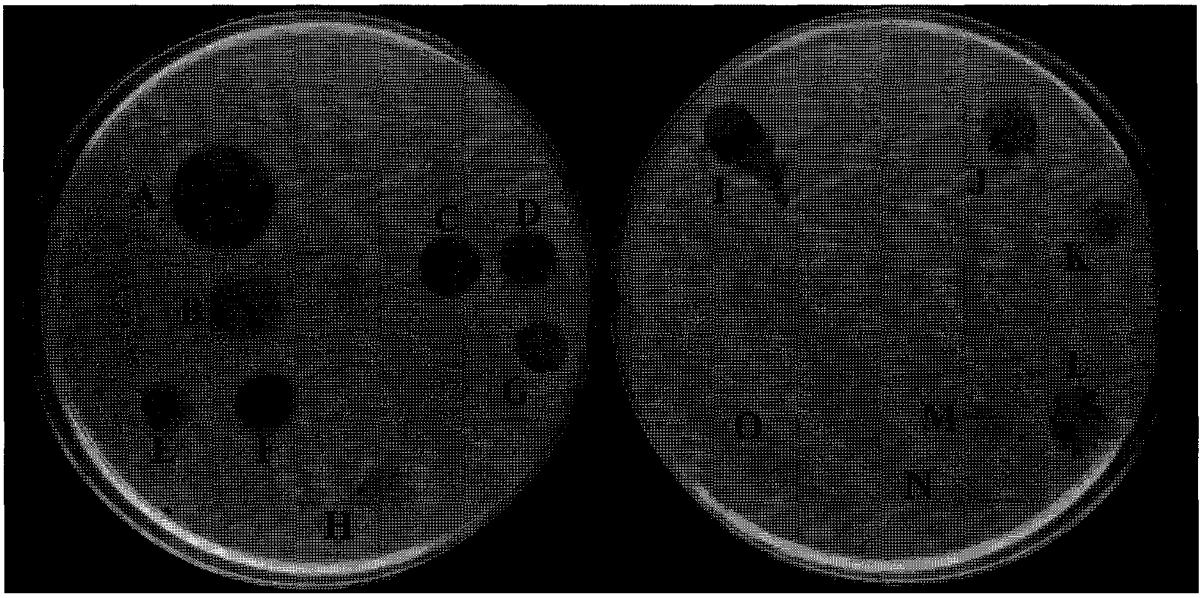

Fig. 1. *Lactobacillus* phage and bacteriocin assay. The indicator strain was *L. delbrueckii* subsp. *lactis* ATCC 15808. Positive inhibitions were shown by lysates from the following strains: **(A)** NCDO 2395, **(B)** L2, **(C)** L1b, **(D)** L1a, **(E)** A1, **(F)** A2, **(G)** Y8, **(H)** Y1, **(I)** Y11, **(J)** Y2, **(K)** Y13, **(L)** Y5, **(M)** Y10a, **(N)** Y10b, and **(O)** Y16. The remaining 23 sample lysates did not lyse ATCC 15808. Each of the small clear plaques in or near a lysis zone is caused by infection of a single bacteriophage. The phage infection or bacteriocin lysis was indicated by a clear lysis zone in the soft agar layer.

plaque assay and by DNA hybridization with the phage $\phi$y8 DNA isolated from a particular brand of yogurt, 11 yogurt strains were identified to release phages. However, only 7 of these yogurt phages lysed the human vaginal *Lactobacillus* strains tested. The rest of the inhibitions were presumably caused by bacteriocins. The bacteriocins from a *Lactobacillus* tablet (L1) and from a yogurt (Y12b) were very potent: both lysed multiple vaginal *Lactobacillus* strains. Bacteriocins isolated from A1, A2, and Y12b were heat stable as they were active against other lactobacilli even after boiling for 10 min. The bacteriocin inhibition pattern of L1 was remarkably similar to that of the type strain of *L. helveticus* NCDO 2395.

Most of the phages were readily identified by direct plaque assay with the indicator strain *L. delbrueckii* subsp. *lactis* ATCC 15808. When a lysis zone displayed multiple small clear plaques within or even adjacent to a turbid lysis zone (Fig. 1), the small plaques were caused by phages. The turbid zone was apparently caused by an antimicrobial activity. The zone disappeared after a 10-fold dilution of the lysate, but the small clear plaques remained. The DNA fingerprinting analysis (data not shown) indicated that 10 phages ($\phi$y1, $\phi$y2, $\phi$y5, $\phi$y8, $\phi$y10, $\phi$y11, $\phi$y12a, $\phi$y13, $\phi$y16, and $\phi$y19) belonged to the same genetic type, although some of these phages had slightly different host ranges (Table 2). We have extensively studied the phage $\phi$y8, released from *L. acidophilus* Y8, the starter strain of a particular brand of yogurt, which was used to treat vaginal yeast infection.[11] The plaque morphology of $\phi$y8 is shown in Figure 1G, and its phage ultrastructural morphology is shown in Figure 2. *L. acidophilus* Y8 cells burst due to spontaneous phage induction at a rate of approximately $10^{-6}$ with a burst size of about 100 phages/cell. The phage $\phi$y8 had a linear double-stranded DNA of about 54 kb. It lysed 4 vaginal *Lactobacillus* strains and 1 dairy strain tested. More detailed data about the phage $\phi$y8 are reported in a separate paper.[15]

### DISCUSSION

Phages and bacteriocins have been well studied in dairy lactobacilli because lactobacilli are important starter cultures for processing dairy foods, especially yogurt.[15] Phages also have been isolated from sewage, sausages, or meat cultures and the human intestine and vagina.[15,17–19] Unlike phages, bacteriocins are non-viable molecules of proteins or peptides. Many lactobacilli have been found to produce bacteriocins of various types.[20] Usually, bac-

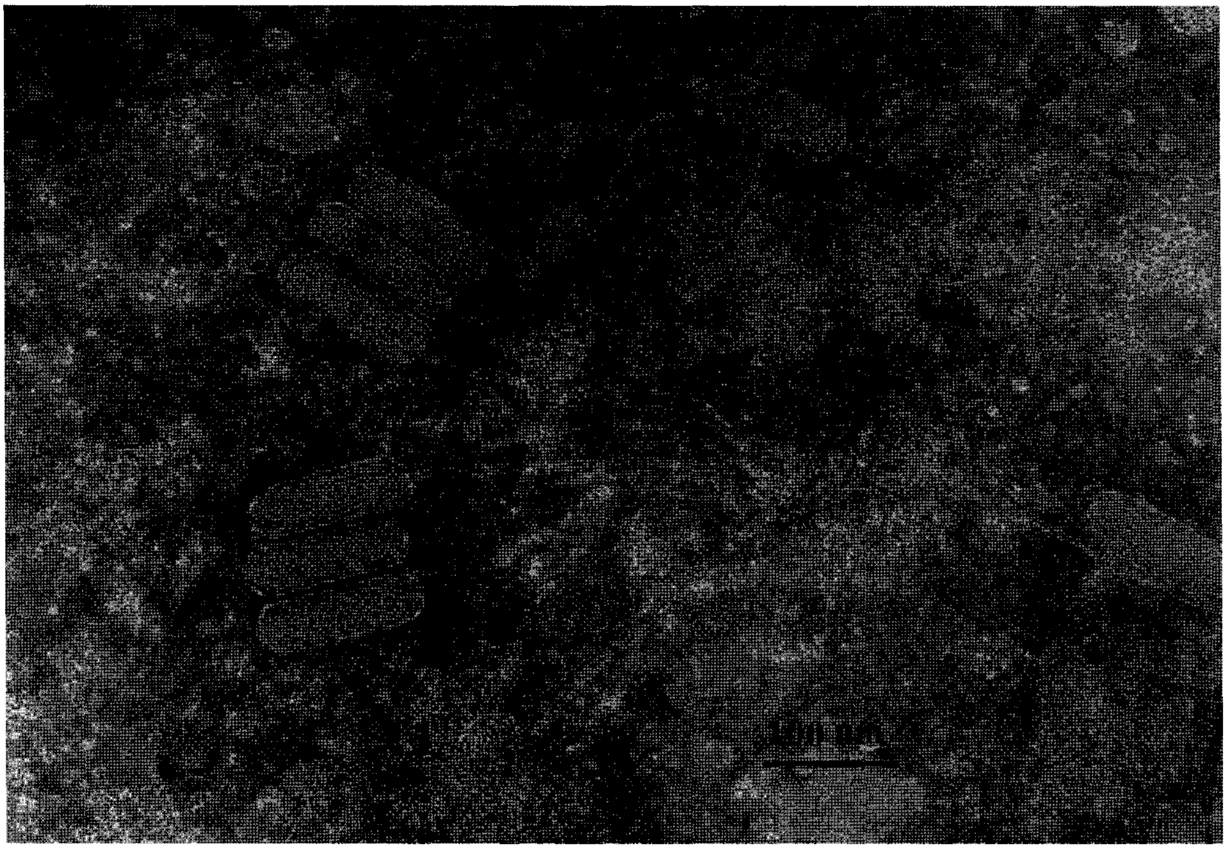

Fig. 2. Electron micrograph of *Lactobacillus* phage ϕy8 from a yogurt.

teriocins made of small peptides can resist heat and/or proteases, while bacteriocins made of large protein molecules can be sensitive to both. The fact that certain commercial *Lactobacillus* products also released phages or bacteriocins was not surprising. It was of interest, however, that some of these phages and bacteriocins lysed human vaginal lactobacilli under in vitro conditions (Table 2). This observation implied that vaginal lactobacilli could be inhibited by *Lactobacillus* phages or bacteriocins from environmental sources.

Unlike antibiotics, which can inhibit a broad spectrum of bacteria, bacteriocins normally only inhibit the same type of bacteria as their producers. Therefore, bacteriocins produced by a *Lactobacillus* strain would most likely inhibit other lactobacilli.[20] While both phages and bacteriocins can inhibit vaginal lactobacilli under in vitro conditions, *Lactobacillus* phages appear to have a relatively narrower host range than bacteriocins. However, a lytic phage may cause greater damage. Once a sensitive *Lactobacillus* strain encounters a virulent phage, the phage can be rapidly reproduced in the bacterial host cells, releasing millions of new phages that can soon eliminate the entire sensitive strain. Conversely, a bacteriocin-producing *Lactobacillus* strain may only cause a limited or a slow adverse effect on a preexistent sensitive strain because bacteriocins cannot be reproduced by target cells and can only kill target cells upon direct contact. Therefore, phage-releasing lysogens can be more virulent than bacteriocin producers in attacking other *Lactobacillus* strains.

We have recently identified that some vaginal lactobacilli release phages, which can efficiently infect the same or other vaginal lactobacilli.[10] Because there is no apparent route for infective vaginal *Lactobacillus* phages to be transmitted among women, it was hypothesized that phages that can infect vaginal lactobacilli may have other sources, such as the environment. Because BV is a common vaginal infection, natural reduction or elimination of vaginal lactobacilli must frequently occur in women.[2–4] However, results from the current study showed that the efficiency of phage infection was not very high—less than 20% of vaginal *Lactobacil-*

*lus* strains tested were sensitive to phages released from dairy lactobacilli. The data suggested that although vaginal lactobacilli can be infected by dairy phages, vaginal lactobacilli might not be a preferred host for these phages, probably due to host-range limitations. Moreover, each day millions of people ingest lactobacilli in various yogurt products, but no apparent side effects have been reported. Therefore, if phages can indeed affect the population of vaginal lactobacilli in vivo in humans, additional environmental phage sources may exist.

A recent study by Antonio and Hillier[21] showed that in many women the vaginal *Lactobacillus* species are identical to their intestinal or rectal *Lactobacillus* species. This observation, together with our earlier observation that a phage released from an intestinal *Lactobacillus* strain lysed multiple vaginal *Lactobacillus* isolates,[10] suggested that intestinal lactobacilli might be a reservoir for phages that infect vaginal lactobacilli. Because the intestine is an open ecological system, intestinal lactobacilli can be originated from various environmental sources, such as food products. Therefore, foods containing lactobacilli that can colonize the intestine and eventually become a part of intestinal microflora may be critical if these lactobacilli release phages that can efficiently infect vaginal lactobacilli.

In summary, phages and bacteriocins released from some *Lactobacillus* products including yogurt, acidophilus milk, tablets, and douche mix inhibited some human vaginal *Lactobacillus* isolates under in vitro conditions. Whereas bacteriocins were isolated from all groups of products studied, phages were isolated only from yogurts. The natural inhibition of vaginal lactobacilli by phages or bacteriocins may be important for studying the initiation of BV because BV is associated with an unexplained decrease of vaginal lactobacilli. Although similar inhibitions observed in vitro may not necessarily occur in vivo in humans, these observations, however, implied a possibility that vaginal lactobacilli might be inhibited naturally by phages or bacteriocins released by lactobacilli from the environment. Further studies will be required to identify additional phage sources and to detect infective phages in vivo in the human vagina.

### ACKNOWLEDGMENTS

We thank Dr. T. Klaenhammer for sending us *L. gasseri* ADH strain and phage ϕadh, S. Robinson and D. Sackuvich for assisting with the electron microscopic study, D. Coats for photography, and P. Puntenney for editing the manuscript.

### REFERENCES

1. Redondo-Lopez V, Cook RL, Sobel JD: Emerging role of lactobacilli in the control and maintenance of the vaginal bacterial microflora. Rev Infect Dis 12:856–872, 1990.
2. Hill GB: The microbiology of bacterial vaginosis. Am J Obstet Gynecol 169:450–454, 1993.
3. Amsel R, Totten PA, Spiegel CA, Chen KCS, Eschenbach D, Holms KK: Nonspecific vaginitis: Diagnostic criteria and microbial and epidemilogical associaton. Am J Med 74:14–22, 1983.
4. Hillier SL, Nugent RP, Eschenbach DA, Krohn MA, Gibbs RS, Martin DH, Cotch MF, Edelman R, Pastorek JG, Rao AV, McNellis D, Regan JA, Carey JC, Klebanoff MA, et al., and Vaginal Infections and Prematurity Study Group: Association between bacterial vaginosis and preterm delivery of a low-birth-weight infant. N Engl J Med 333:1737–1742, 1995.
5. Hallen A, Jarstrand C, Pahlson C: Treatment of bacterial vaginosis with lactobacilli. Sex Transm Dis 19:146–148, 1992.
6. Hillier SL, Krohn MA, Klebanoff SJ, Eschenbach DA: The relationship of hydrogen peroxide-producing lactobacilli to bacterial vaginosis and genital microflora in pregnant women. Obstet Gynecol 79:369–373, 1992.
7. Klebanoff SJ, Hillier SL, Eschenbach DA, Waltersdorph AM: Control of the microbial flora of the vagina by $H_2O_2$-generating lactobacilli. J Infect Dis 164: 94–100, 1991.
8. Harwood B, Mittendorf R, Judge D, Dayal S, Walker C: Patterns of vaginal douching and their association with vaginal bacteriosis. Infect Dis Obstet Gynecol 4:51, 1996.
9. Hooton TM, Fennell CI, Clark AM, Stamm WE: Nonoxynol-9: Differential antibacterial activity and enhancement of bacterial adherence to vaginal epithelial cells. J Infect Dis 164:1216–1219, 1991.
10. Pavlova SI, Kiliç AO, Mou SM, Tao L: Phage infection in vaginal lactobacilli: An in vitro study. Infect Dis Obstet Gynecol 5:36, 1997.
11. Hilton E, Isenberg HD, Alperstein P, France K, Borenstein MT: Ingestion of yogurt containing *Lactobacillus acidophilus* as prophylaxis for candidial vaginitis. Ann Intern Med 116:353–357, 1992.
12. Hughes VL, Hillier SL: Microbiologic characteristics of *Lactobacillus* products used for colonization of the vagina. Obstet Gynecol 75:244–248, 1990.
13. Kandler O, Weiss N: Genus *Lactobacillus*. In Sneath P (ed): Bergey's Manual of Systematic Bacteriology. Vol 2. Baltimore: Williams & Wilkins, pp 1209–1234, 1986.
14. Bradley DE: Ultrastructure of bacteriophage and bacteriocin. Bacteriol Rev 31:230–314, 1967.
15. Kiliç AO, Pavlova SI, Ma W, Tao L: Analysis of *Lacto-*

*bacillus* phages and bacteriocins in American dairy products and characterization of a phage isolated from yogurt. Appl Environ Microbiol 62:2111–2116, 1996.

16. Maniatis T, Fritsch EF, Sambrook JJ: Molecular Cloning C: A Laboratory Manual. Cold Spring Harbor, NY: Cold Spring Harbor Laboratory Press, 1982.
17. Kopeloff N: Dissociation and filtration of *Lactobacillus acidophilus*. J Infect Dis 55:368–372, 1934.
18. Nes IF, Sorheim O: Effect of infection of a bacteriophage in a starter culture during the production of salami dry sausage: A model study. J Food Sci 49:337–340, 1984.
19. Raya RR, Kleeman EG, Luchansky JB, Klaenhammer TR: Characterization of the temperate bacteriophage φadh and plasmid transduction in *Lactobacillus acidophilus* ADH. Appl Environ Microbiol 55:2206–2213, 1989.
20. Dodd HM, Gasson MJ: Bacteriocins of lactic acid bacteria. In Gasson MJ, De Vos WM (eds): Genetics and Biotechnology of Lactic Acid Bacteria. London: Chapman & Hall, 1994.
21. Antonio M, Hillier SL: The identification of *Lactobacillus* species in the rectum of women. 96 General Meeting of American Society for Microbiology, Abstract C394, 1996.
